# Supplementary material for: “It really proves to us that we are still valuable”: Qualitative research to inform a safer conception intervention for men living with HIV in South Africa
Source: PLoS One. 2021 Mar 25;16(3):e0240990. doi: 10.1371/journal.pone.0240990 (PMC7993862; doi:10.1371/journal.pone.0240990)
Supplement: S1 File — (PDF) [file pone.0240990.s001.pdf]

# Helping Men Have Healthy Babies

| FOCUS GROUP ID NUMBER: | DATE (DD/MMM/YY): | START TIME: | END TIME: | FACILITATOR INITIALS: |
|---|---|---|---|---|
| | | | | |

**[Read to participants]**

We are doing a study to develop a counseling programme to help men who have HIV to have babies as safely as possible. You have been invited here today to talk about this programme.

We have just reviewed the consent forms, which describe the study in detail and gives us permission to speak with you. As a reminder, you are not required to answer all of my questions, and you may skip any questions. As a reminder, I will use a digital recorder to record our conversation. Do you have any questions before we begin the discussion?

*Senza ucwaningo ukuthuthukisa uhlelo lokweluleka ukusiza ebesilisa abanegciwane lesandulela ngculaza(HIV) bathole izingane ngendlela ephephe kahle. Umenyiwe lana namuhla ukukhuluma ngaloluhlelo.*

*Sisanda kubukeza ifomu lokuvuma, elichaza ucwaningo kabanzi futhi lisinikeza imvume yokukhuluma nawe. Njenge sikhumbuzo, awulindelekile ukuphendula yonke imibuzo yami, futhi ungeqa namiphi imibuzo. Njenge sikhumbuzo, ngizosebenzisa isiqophamazwi ukuqopha ingxoxo yethu. Ingabe ninayo imibuzo ngaphambi kokuba siqale ingxoxo?*

**Turn on digital recorder.**

I am (FACILITATOR NAME) interviewing (FOCUS GROUP ID#) on [DATE] [START TIME]

*Ngingu (IGAMA LOMPHATHI NGXOXO ) ngibuza (INGXOXO YEQEMBU ID#) ngo [SUKU] [ISIKHATHI SOKUQALA]*

| | **Main question/ *Umbuzo omkhulu*** | **Probe/ *Buzisisa*** |
|---|---|---|
| 1. | We know that many men living with HIV want to have children, yet very few receive information on how to have children without infecting their partner or future child.<br><br>*Siyazi ukuthi abesilisa abaphila negciwane lesandululela ngculaza bayafuna ukuba nezingane, noma kunjalo bancane kakhulu abathola ulwazi lokuthi bangazithola kanjani izingane ngaphandle kokuthelela ophathina babo noma umntwana ngokuzayo.* | a. What do you know about this? *(Yini oyaziyo mayelana nalokhu?)*<br>b. Have you heard of any strategies for keeping your partner HIV-uninfected while having children? (*Ingabe uke wezwa nganoma imaphi amasu okuthi ungamgcina kanjani uphathina wakho engathelelekile ngegciwane lesandululela ngculaza (HIV) ngesikhathi ethola ingane?)*<br>*c.* Have you heard of any strategies for having an HIV-uninfected baby? (*Ingabe uke wezwa nganoma imaphi amasu okuthola ingane engathelelekile ngegciwane lesandulela ngculaza HIV?)* |
| 2. | Because there are many steps to having an uninfected child, this programme would require men to attend 3 counseling sessions. These sessions would be one-on-one meetings and would provide information about what HIV positive men can do to have an uninfected child.<br><br>*Ngenxa yokuba kunezinyathelo eziningi ukuba nengane engathelelekile, loluhlelo luzodinga abesilisa ukuba bahambele imihlangano emi 3 yokwelulekwa. Le mihlangano izokuba imihlangano yomuntu nomuntu futhi iyonikeza ulwazi mayelana nokuthi yini owesilisa onegciwane lesandulela ngculaza angayenza ukuba nengane engathelelekile* | a. How do you think attending 3 sessions might work for you or other men from this clinic? (*Ucabanga ukuthi ukuhambela imihlangano emi 3 bekungakusebenzela kanjani wena noma abanye abesilisa kulomtholampilo?)*<br>b. Would you or other men be able to attend these meetings? (*Wena noma abanye abesilisa bebengakwazi ukuhambela lemihlangano?)*<br>c. What would make it difficult to attend? (*Yini ebingakwenza kubenzima ukuhambela?)*<br>d. What times would be good for meeting? (*Yiziphi izikhathi ebezingaba ezilungele umhlangano?)*<br>e. Would you prefer to meet at the clinic or at another site? (*Ubungancamela ukuhlanganela emtholampilo noma kwenye indawo?)* |

| | | |
|---|---|---|
| 3. | Women also have an important role to play in having HIV uninfected children. How do you think men like you would feel about the possibility of bringing the partner who they want to have a child with to one or more of the meetings?<br><br>*Abesifazane nabo banendima ebalulekile ukuyidlala ukuthola izingane ezingathelelekile ngegciwane lesandulela ngculaza (HIV). Ucabanga ukuthi abesilisa abanjengawe bebengazizwa kanjani mayelana nokuletha uphathina abafuna ukuba nengane naye kowodwa noma emihlanganweni eminingi?* | a. Would that work for someone like you? (*Kungasebenza lokho kumuntu onjengawe?)*<br>b. Why or why not? (Tell me more.) (*Kungani noma kungani kungenjalo? (Ngitshele okunye.))* |
| 4. | I want to share with you some of the safer conception strategies that are available and ask your opinions about whether these could work for HIV-infected men here in Umlazi. Let's say that Sipho is an HIV infected man and he wants to have a child with his partner Naledi. Naledi does not have HIV, and one of the ways to make sure Sipho has an uninfected baby is to protect Naledi from HIV.<br><br>*Ngifuna ukwabelana nawe ngamanye amasu aphephile okukhulelwa akhona futhi ngibuze uvo lwakho mayelana nokuthi ingabe angasebenza kwabesilisa abatheleleke ngegciwane lesandulela ngculaza (HIV) lapha eMlazi. Ake sithi uSipho ungowesilisa otheleleke ngegciwane lesandulela ngculaza (HIV) futhi ufuna ukuba nengane nophathina wakhe uNaledi. uNaledi akanalo igciwane lesandulelela ngculaza, futhi enye indlela ukwenza isiqinisekiso sokuthi uSipho unengane engathelelekile, ukuvikela uNaledi kwigciwane lesandulela ngculaza (HIV).* | |
| 4a. | Naledi is more likely to get pregnant during particular times in her menstrual cycle. One way to reduce the risk of transmitting HIV to Naledi while trying to get pregnant is to use condoms most of the time. But when Naledi is most fertile, the couple can have sex without condoms to try to conceive. This method is known as "**timed intercourse**" and can be used to reduce the risk of HIV transmission between partners, while still allowing the woman to become pregnant.<br><br>*UNaledi kungenzeka kakhulu akhulelwe ngezikhathi ezithile zokuya esikhathini(menstrual cycle). Indlela eyodwa ukunciphisa ubungozi bokudlulisela igciwane lesandulela ngculaza (HIV) kuNaledi ngesikhathi ezama ukukhulelwa ukusebenzisa amakhondomu isikhathi esiningi. Kepha ngesikhathi uNaledi ekulungele ukukhulelwa (fertile), ababili bangalwenza* | a. What do you think of this method? (*Ucabangani ngalendlela?)*<br>b. Would it work for you? (*Ibingasebenza yini kuwena?)*<br>c. What do you think your partner would think of this method? (*Yini ocabanga ukuthi uphathina wakho ube ngayicabanga ngale ndlela?)*<br>d. How would this work for other men? Why do you think this? (*Ibingasebenza kanjani kwabanye besilisa?)* |

| | | |
|---|---|---|
| | *ucansi ngaphandle kwamakhondomu ukuzama ukukhulelwa (conceive). Lendlela yaziwa ngocansi olubekelwe isikhathi ("**Timed intercourse")** futhi ingase tshenziswa ukunciphisa ingozi yokudlulisela igciwane lesandulela ngculaza(HIV) phakathi kophathina, ngesikhathi evumela owesifazane ukuthi akhulelwe.* | |
| 4b. | If Sipho is started on antiretrovirals (ARVs) and takes his pills every day, the level of HIV virus in his blood will be very low. When the HIV virus in his blood is low, it is unlikely that Sipho can transmit the virus to Naledi or to their baby. This is sometimes called **treatment as prevention**.<br><br>*Uma uSipho ubeqaliswa ngemishanguzo yesandulela ngculazi (ARVs) futhi ethatha amaphilisi akhe nsuku zonke, izinga legciwane lesandulela ngculaza egazini lakhe liyoba phansi kakhulu. Uma igciwane lesandulela ngculaza egazini lakhe liphansi, akulula ukuthi uSipho angadlulisela igciwane kuNaledi noma enganeni yabo. Lokhu kubizwa **ukulashw**a **njengokuvikela** – (**treatment as prevention).*** | a. What do you think of this method? *(Yini oyicabangayo ngalendlela?)*<br>b. Even though your CD4 count is high and you wouldn't normally qualify for treatment, what would it be like for you to start treatment early, to have an uninfected baby? (*Noma ngabe amasosha akho omzimba ephezulu futhi ungakakulungeli ukuqala imishanguzo, bekunganjani kuwena ukuqala imishanguzo masisha, ukuze ube nengane engathelelekile?)*<br>c. Would it work for you? (*Ibingakusebenzela wena?)*<br>d. What do you think your partner would think of this method? (*Yini ocabanga ukuthi uphathina wakho ubengayicabanga ngalendlela?)*<br>e. How would this work for other men? Why do you think this? (*Ibingabasebenzela kanjani abanye besilisa? Kungani ucabange lokhu?)* |
| 4c. | Another option, which is still being studied, would be for Naledi – who is uninfected – to take a kind of ARVs called **PrEP or pre-exposure prophylaxis**. With PrEP, an HIV-negative partner with an HIV-positive partner can take certain ARVs in order to reduce the chances of getting HIV. Doctors are still studying whether this method works and how PrEP might be used for couples who want to have a baby.<br><br>*Enye indlela, esacwaningwa, eyokuba uNaledi ongathelelekile – athathe uhlobo lwemishanguzo ebizwa **i-PrEP, i-pre-exposure prophylaxis**. Nge PrEP, uphathina ongenalo igciwane lesandulela ngculaza onophathina* | a. What do you think of this method? (*Yini oyicabangayo ngalendlela?)*<br>b. Would it work for you? (*Ibingakusebenzela wena?)*<br>c. What do you think your partner would think of this method? (*Yini ocabanga ukuthi uphathina wakho ubengayicabanga ngalendlela?)*<br>d. How would this work for other men? (*Kungabasebenzela kanjani lokhu abanye besilisa?)*<br>e. Why do you think this? (*Kungani ucabange lokhu?)* |

| | | |
|---|---|---|
| | *onegciwane lesandulela ngculaza angathatha imishanguzo (ARVs) ethile ukwenzela ukunciphisa amathuba okuthola iHIV. ODokotela basacwaninga ukuthola ukuthi lendlela iyasebenza yini nokuthi iPrEP ibingasetshenziswa kanjani ophathina abafuna ukuthola ingane.* | |
| 4d. | Have you heard of any other methods that might work for this couple?<br><br>*Usuke wezwa ngezinye izindlela ezingasebenza ngalaba ababili?* | |
| 5. | When we are ready to study the effects of the counseling programme, we will be interested in the timing of sex. Therefore, we would want to ask men in the study to report on their sexual behavior frequently. We would do this by sending an SMS to a cell phone to ask a few brief yes/no questions about sexual practices that day. Sample questions would be, “Did you have sex today?” and “Did you use a condom?”<br><br>*Uma sesikulungele ukucwaninga ngemithelelayohlelo lokweluleka, siyothanda ukubheka ukucansi oluhlelelwe (‘timing of sex’). Ngakho ke, singathanda ukucela abesilisa abasocwaningweni ukuba babike ngokuziphatha kwabo ngokocansi kaningana . Siyokwenza lokhu ngokuthumela iSMS kumakhalekhukhwini ukubuza imibuzo embalwa emifushane engo yebo/cha imibuzo mayelana nezindlela zokwenza ucansi ngalelolanga. Isibonelo semibuzo: “Ulwenzile ucansi namuhla?” futhi “Ingabe uyisebenzisile ikhondomu?”* | a. How do you think this would work for men like you? (*Ucabanga ukuthi kuzosebenza kanjani lokhu kwabanye besilisa abanje ngawe?)*<br>b. What might get in the way of this? (*Yini engama endleleni yalokhu?)*<br>c. Some people might worry about privacy. If the text were protected with a password, how would that influence your feelings about this? (*Abanye abantu bangakhathazeka ngendaba yobumfihlo. Uma okubhaliwe bekuvikelekile nge (password), imizwa yakho ibingathikamezeka kanjani mayelana ngalokhu?)* |
| 6. | We are studying this counseling programme in order to see if it makes a difference in men’s ability to have uninfected children. To study the program, half of the men in the study will participate in the safer conception counseling programme and the other half will complete a different counseling program. The group that does not get the safer conception counseling will be part of a jobs training programme. At the end, both groups will get to learn a bit of what was taught in the other group. | a. What sort of job training would be helpful to men like you in Umlazi? (*Ukuqeqeshelwa umsebenzi onjani obekunga wusizo kwabesilisa abanjenganawe eMlazi?)* |

| | | |
|---|---|---|
| | *Sicwaninga loluhlelo lokweluleka ukubona ukuthi liyawenza umehluko kwabesilisa bakwazi ukuba nezingane ezingathelelekile. Ukucwaninga loluhlelo, ingxenye yabesilisa abasocwaningweni iyobamba iqhaza ohlelweni lokwelulekwa lokukhulelwa oluphephile kantii enye ingxenye iyogcwalisa uhlelo lokwelulekwa olwehlukile. Iqembu elingatholi ukwelulekwa kokukhulelwangendlela ephephile liyokuba ingxenye yohlelo lokuqeqeshelwa imisebenzi. Ekugcineni, amaqembu omabili, ayothola ukufunda okuncane obekukade kufundiswa kwelinye iqembu.* | |
| 7. | Do you have any additional questions?<br><br>*Ingabe unayo eminye imibuzo?* | |

I want to take this opportunity to remind you that the way babies acquire HIV is through the mother. So the best way to help insure that you have an uninfected child is to make sure that the mother is uninfected and remains uninfected at conception and during the pregnancy. Or, if the mother is HIV infected, then she should be in care and on antiretroviral therapy in order to reduce the chances of her transmitting HIV to the baby during pregnancy, childbirth, and breastfeeding.

Thank you for your participation.

*Ngithanda ukuthatha lelithuba ukukukhumbuza ukuthi indlela izingane ezithola ngayo igciwane lesandulela ngculaza(HIV) zilidluliselwa ngumama. Ngakho-ke indlela engcono ukusiza ukuqinisekisa ukuthi unengane engathelelekile ukwenza isiqiniseko sokuthi umama akathelelekile futhi uhlala engathelelekile lapho ekhulelwa nangesikhathi esekhulelwe. Noma, uma umama engciwane lesandlulela ngculaza , kumele anakekeleke futhi abe ngothatha imishanguzo yesandulela ngculaza (ARVs) ukwenzela ukunciphisa amathuba akhe okudlulisela igciwane lesandulela ngculaza(HIV) enganeni ngesikhathi ekhulelwe, ebeletha, futhi encelisa.*

*Ngiyabonga ngokubamba kwakho iqhaza.*
