# Supplementary material for: “It really proves to us that we are still valuable”: Qualitative research to inform a safer conception intervention for men living with HIV in South Africa
Source: PLoS One. 2021 Mar 25;16(3):e0240990. doi: 10.1371/journal.pone.0240990 (PMC7993862; doi:10.1371/journal.pone.0240990)
Supplement: Attachment — Submitted filename: Response to Reviewers.pdf [file pone.0240990.s002.pdf]

Reviewer #1: I wish to thank the authors for the excellent job that they have done in revising the manuscript. It has now become stronger especially the Results and Discussion sections.

I was pleased to note that the study limitations including those of the data used in this paper have now been expanded and discussed in the Discussion section.

My comments I raised in the previous round of reviews have been adequately addressed.

Thank you.

Response. Thank you for the helpful comments and for reviewing the revision.

Reviewer #2: Thank you for your paper it is a very important topic which is not widely written about, and the paper is very well written. Nonetheless, I offer a few thoughts below which I hope resonate and may enrich the paper further.

I do have a small concern that the data is from 2014, HIV knowledge and treatment access has shifted significantly in this time – how might this shift outcomes? I see that the authors address this in the discussion, however as the reader until I get to the discussion this issue was 'shouting very loudly at me'. Perhaps you could briefly acknowledge this earlier on and then discuss it in detail in the discussions section?

Response. Thank you for this comment. We added the following to the introduction: "While these data were collected in 2014, men living with HIV in South Africa remain an under-reached population, their reproductive goals are not yet integrated into HIV care, and we believe these data remain relevant to understanding the needs and considerations of men living with HIV in South Africa in 2020."

Abstract: I have a few points here, please apply appropriately throughout the article as well.
"Twelve participants from three FGDs had a median age of 37 (range 23-45) years, reported a median of 2 (range 1-4) sexual partners, and 1 (range 1-3) desired pregnancy partner(s). A third (N=4) had disclosed HIV-serostatus to the pregnancy partner. " this is a bit confusing, what does 'desired pregnancy partners' mean? And please explain a 'pregnancy partner'. This comes up in Table 1 as well, please clarify the term, I assume it means they have a partner who they would want to conceive with?

Response. This study enrolled men planning for a pregnancy with a female partner in the next year. Therefore we asked questions about the enrolled men's desired pregnancy partner. We added additional clarification of this term where first used in the methods and the abstract.

"Conclusions: Men living with HIV are interested in safer conception and willing to attend clinic programs." please think about whether you can generalize to 'Men living with HIV…" as you have done here – qualitative research provides incredibly important information, however, there are many reasons why we tend not to generalize in this way. Perhaps re-phrase to "These men living with…"

Response. - We have edited this to ensure that readers understand that abstract conclusions refer to this data in the manuscript. This sentence now reads, "Men living with HIV expressed interest in safer conception and willingness to attend clinic programs…".

Selection of partners: I assume they were receiving care in the public health sector? Please specify.

Also, “Potential participants were recruited at a facility 69 based in KwaZulu-Natal” – again I assume a public facility? Please specify.

Response. In the methods section we indicated that men were recruited from an NGO/Department of Health (DoH) collaborative healthcare facility based in a large township in eThekwini, KwaZulu-Natal, South Africa. We did not recruit the partners of the men for the focus group discussions.

Data analysis: “Quantitative data from the questionnaires are described.” please explain in more detail.

Response. We have amended this sentence which now reads:
“Quantitative data from the questionnaires are described using median (range) and number (%).”

Results: the authors note that “When men were encouraged to think of ways to protect a partner from HIV while conceiving they described sperm washing, in vitro fertilization, and donor sperm as strategies they had heard of but did not know how to access.” It is very interesting, and quite surprising, that they had heard of these methods, but not the other methods – could you perhaps indicate how many had heard of it, to give a fuller picture, and perhaps also a comment about how much they knew and how accurate their understanding was.

Response. We added additional language to indicate what men shared about sperm washing. Because this is a qualitative paper with few men and we, therefore, did not systematically assess what all men knew about sperm washing (or any of the methods), we would prefer not to list the number of men who appeared to understand sperm washing as this is not really well aligned with the methods used in this manuscript.

Discussion: in your first paragraph you include the challenge of recruiting men and limited hours of facilities etc. While this is an important point, I don’t think it should be in this first paragraph, in my view it is not the most important point and should not be at the start of your discussion. I would shift it elsewhere.

Response. Thank you for this suggestion. We have moved this discussion to a later section on the challenges of engaging men.

You mention that “This is supported by the first demonstration project of safer conception services in South Africa wherein all men were able to engage their partners”, please specify when this was conducted and how many men were involved, to provide more context and understanding for the reader.

Response. Thank you for this suggestion. We have added more details about this project.

“PLWH do not ask about options for pregnancy for many reasons, including sensing that providers are not supportive of their reproductive goals” – can you expand on this? Is this stigma around PLWH or what?

Response. We have edited this sentence which now reads:
“PLWH do not ask about options for pregnancy for many reasons, including sensing that providers are not supportive of their reproductive goals and stigma towards PLWH having children (4, 16, 40).”

Thank you this was an interesting read.
Response. Thank you for your helpful review!
